# Supplementary figures and images for: Effects of Inbreeding on Microbial Community Diversity of Zea mays
Source: Microorganisms. 2023 Mar 29;11(4):879. doi: 10.3390/microorganisms11040879 (PMC10145435; doi:10.3390/microorganisms11040879)

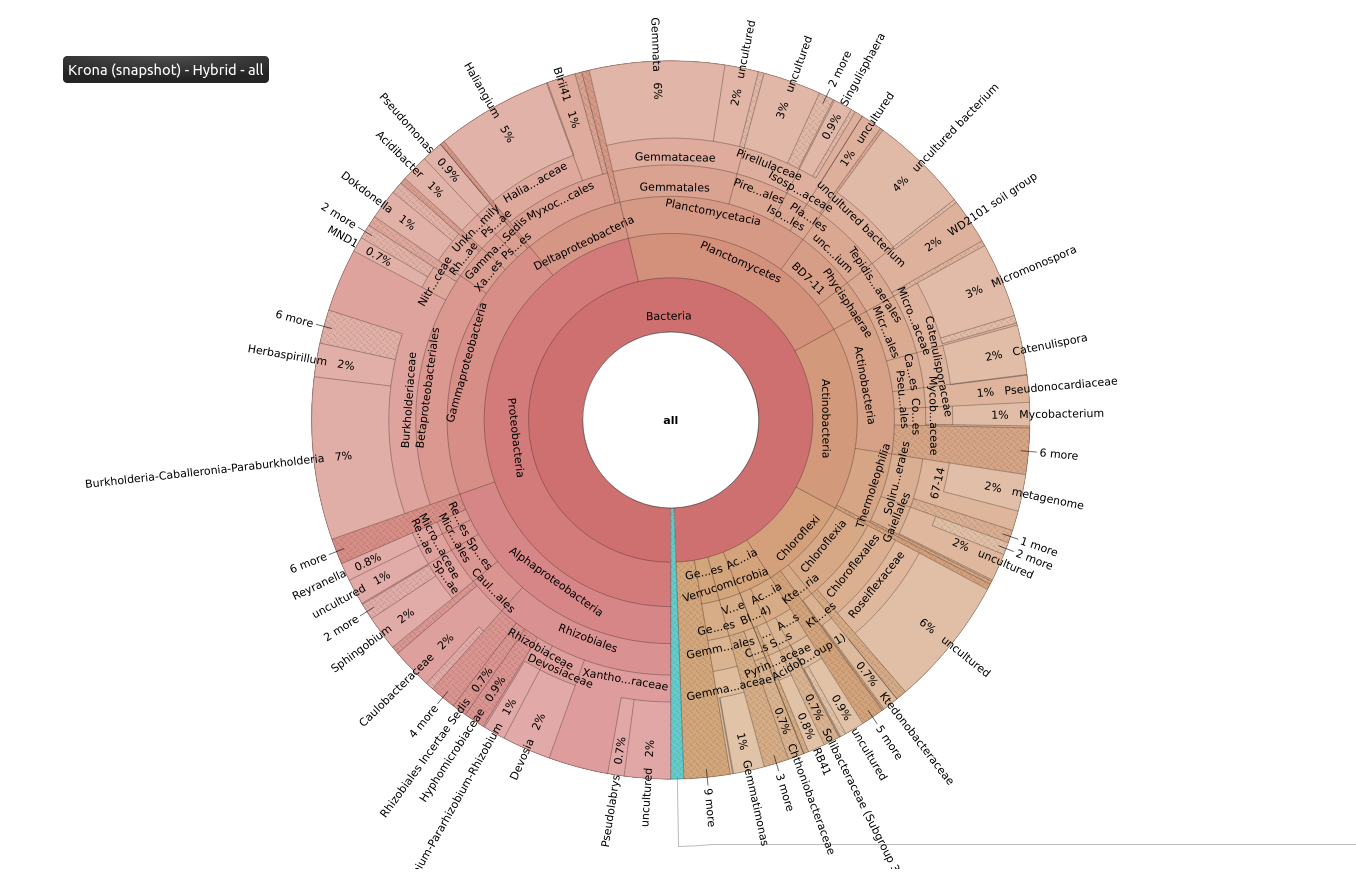

Supplement: Supplementary file 1 [file microorganisms-11-00879-s001.zip › DatasetS1_KronaPlots/Roots_Hybrid_Krona.png]

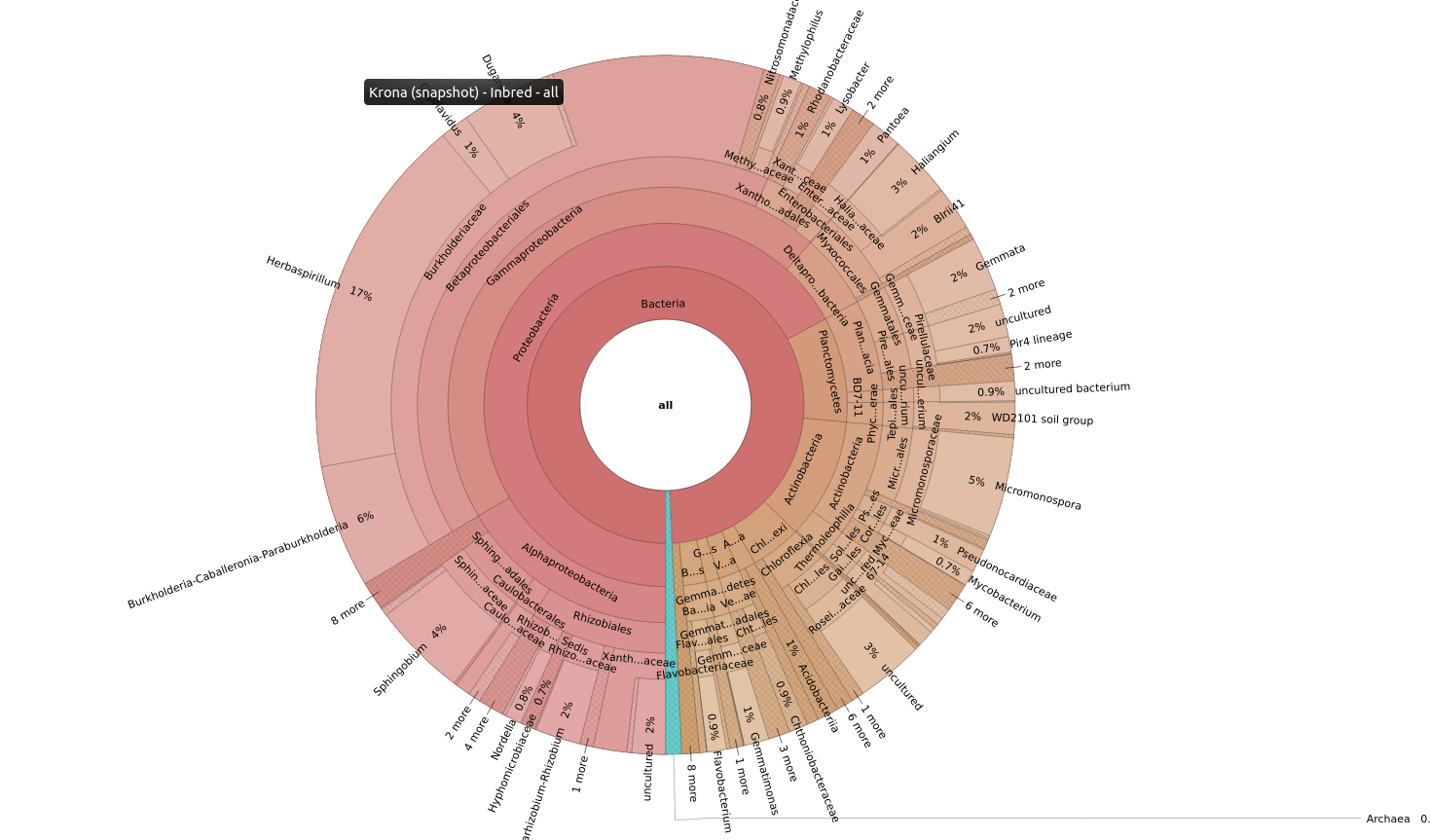

Supplement: Supplementary file 1 [file microorganisms-11-00879-s001.zip › DatasetS1_KronaPlots/Roots_Inbred_Krona.png]

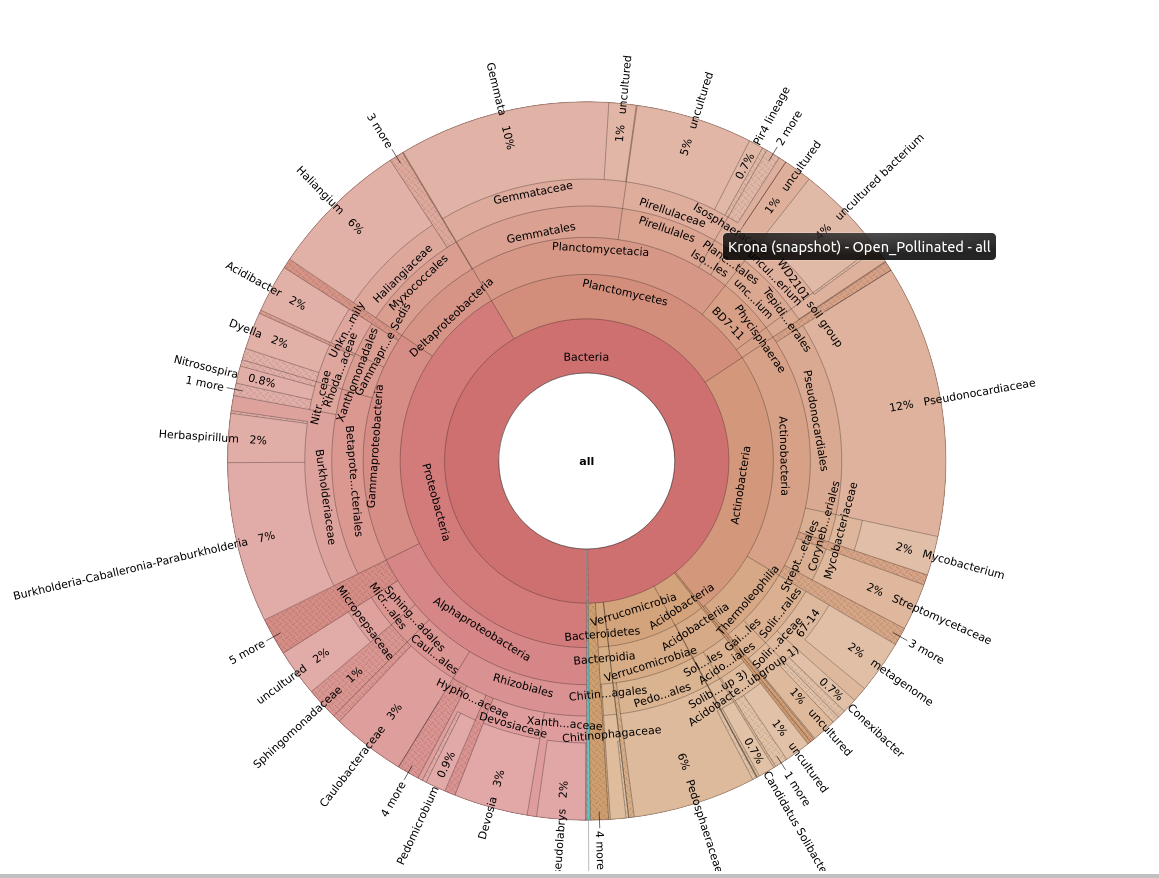

Supplement: Supplementary file 1 [file microorganisms-11-00879-s001.zip › DatasetS1_KronaPlots/Roots_OpenPol_Krona.png]

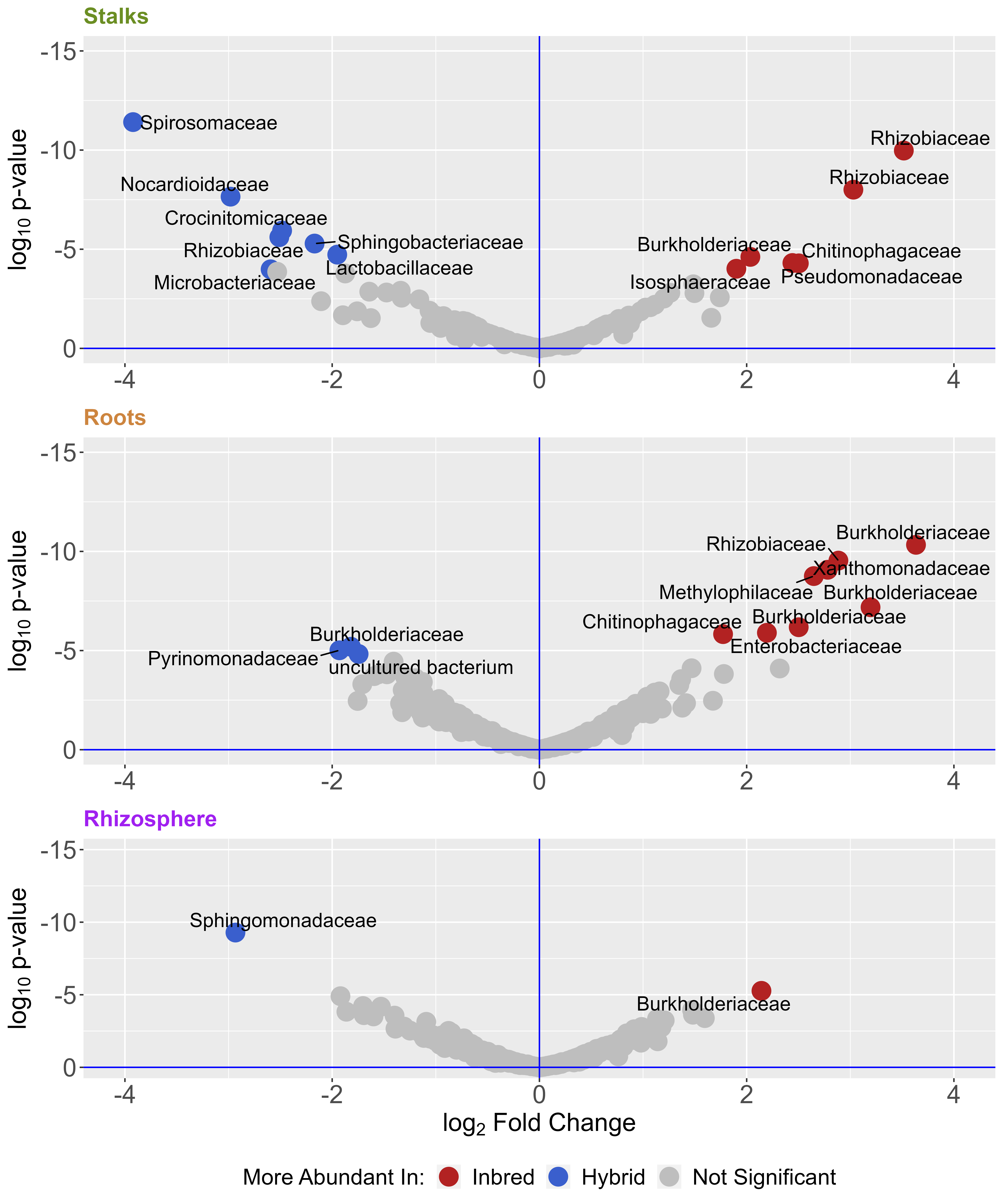

Supplement: Supplementary file 1 [file microorganisms-11-00879-s001.zip › DatasetS2_DiffAbunTables/Fig4_IvsH_D4.png]

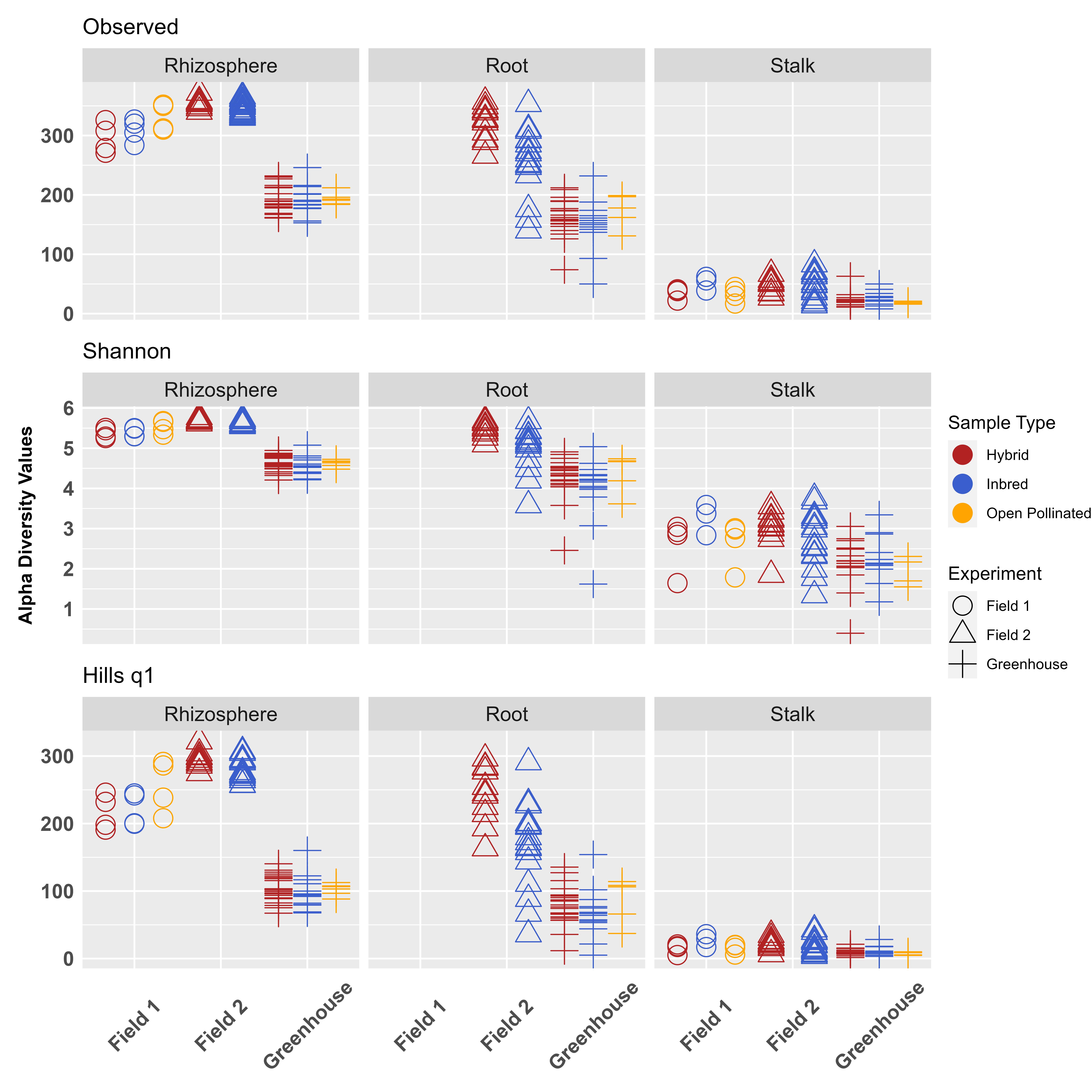

Supplement: Supplementary file 1 [file microorganisms-11-00879-s001.zip › FigS1_Alpha_Revised.png]

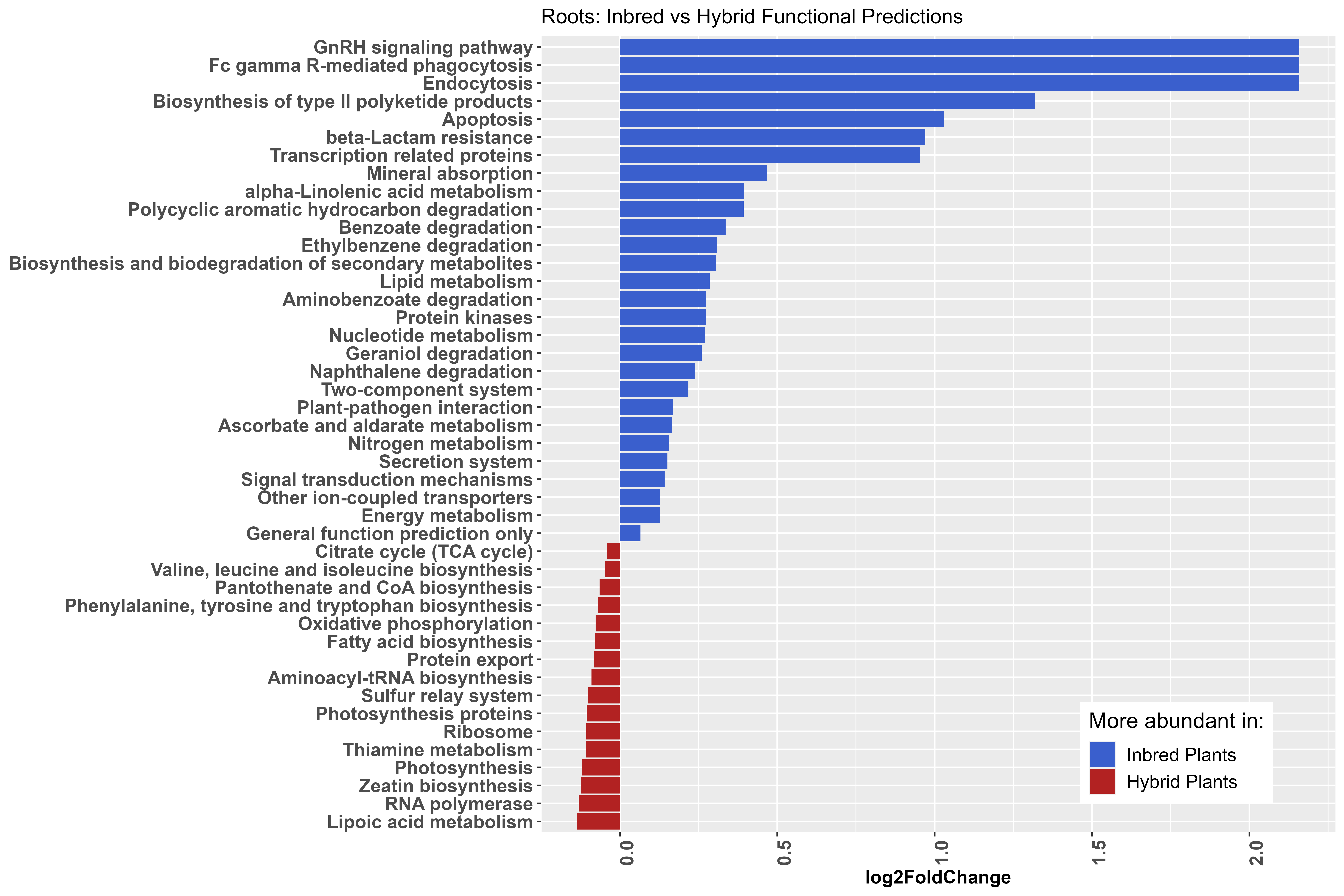

Supplement: Supplementary file 1 [file microorganisms-11-00879-s001.zip › FigS2_IvHpicrust_Root_Revised.png]

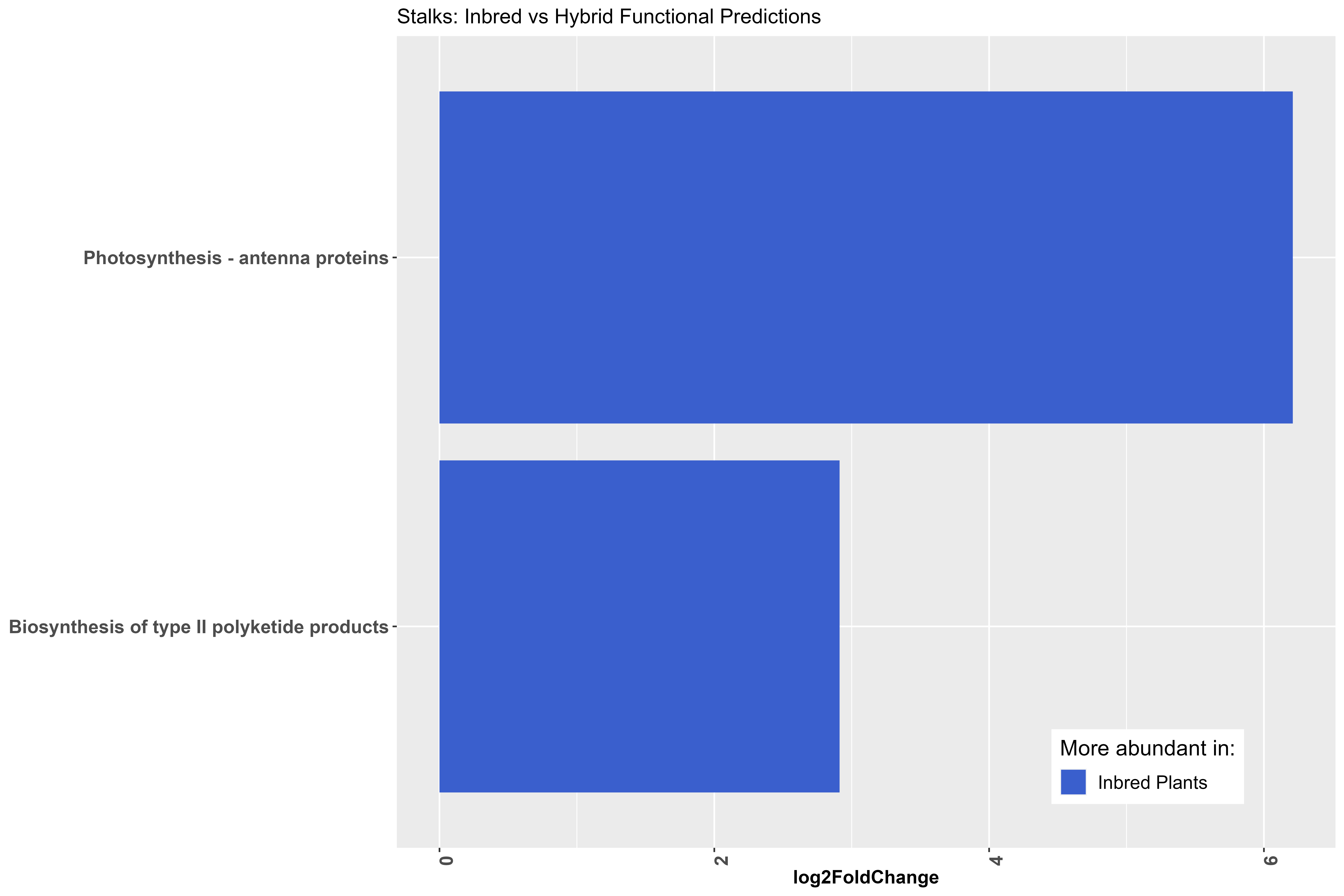

Supplement: Supplementary file 1 [file microorganisms-11-00879-s001.zip › FigS2_IvHpicrust_Stalk_Revised.png]

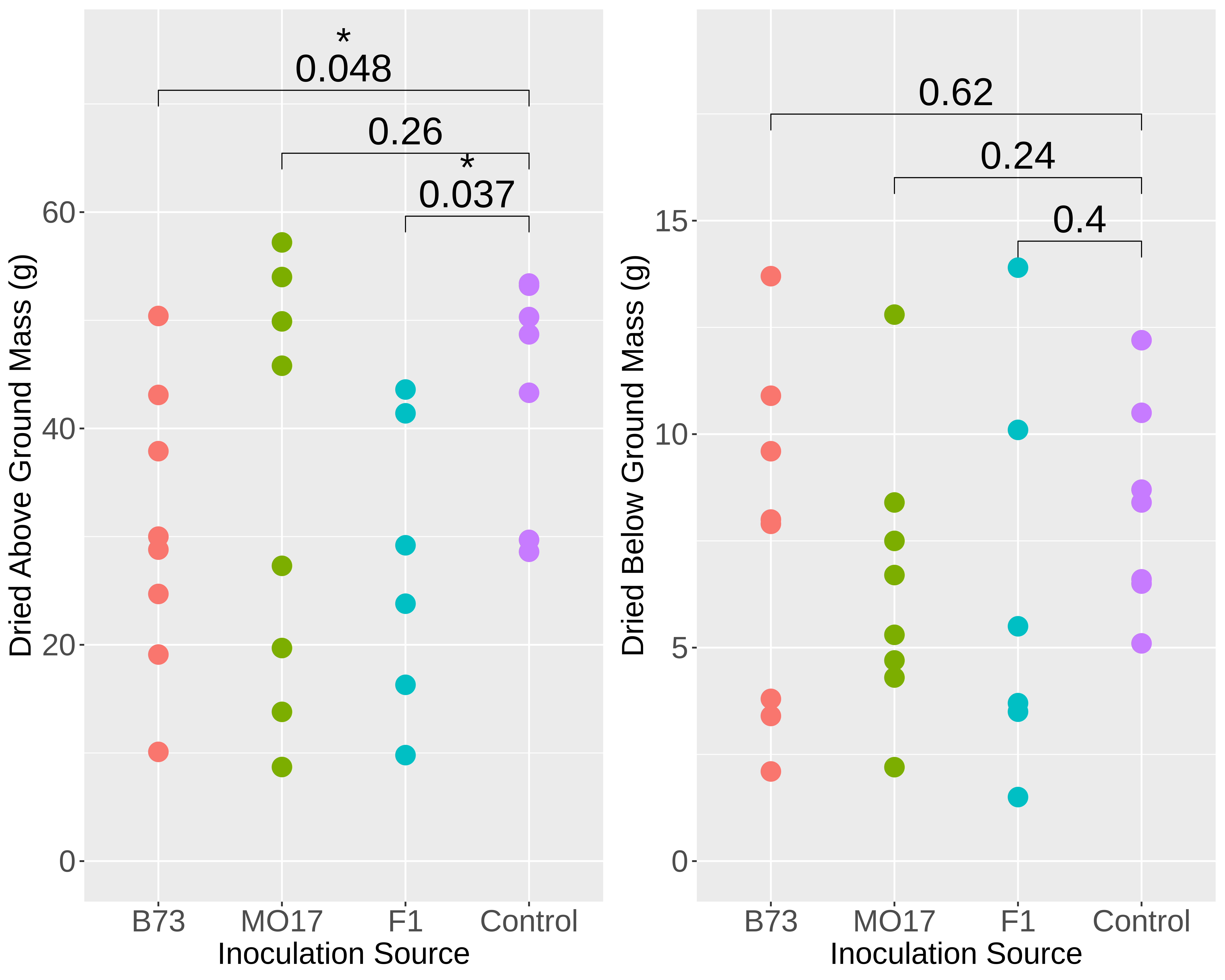

Supplement: Supplementary file 1 [file microorganisms-11-00879-s001.zip › FigS3_MMinoc.png]
